# “Where are we on the Road Towards Family-focused Practice in Mental Healthcare?” - Perspectives from a Swedish/Norwegian Research Collaborative

**DOI:** 10.1007/s10597-025-01499-0

**Published:** 2025-08-25

**Authors:** Lisbeth Kjelsrud Aass, Bente Weimand, Mats Ewertzon, Hege Skundberg-Kletthagen, Ingrid Lindholm, Øyfrid Larsen Moen, Agneta Schröder, Nina Beate Andfossen

**Affiliations:** 1 Department of Health Sciences, Norwegian University of Science and Technology, Gjøvik, Norway; 2 Department of Health, Social and Welfare Studies, University of South-Western Norway, Drammen, Norway; 3 Clinical Mental Health and Addiction Division, Akershus University Hospital, Lorenskog, Norway; 4 The Swedish National Family Care Competence Centre, Kalmar, Sweden; 5 University Health Care Research Center, Faculty of Medicine and Health, Örebro University, Örebro, Sweden

**Keywords:** Caring roles, Family-focused practice, Mental health, Volunteers

## Abstract

As a Norwegian/Swedish research network based on a family-focused practice in mental healthcare, we recognise the need to highlight this approach for the future quality and sustainability of the care and services provided. The role of family members in caring for individuals with mental health conditions is situational and diverse, encompassing several support areas such as emotional support, continuation of social and living skills, economic assistance, and monitoring for signs of illness and relapse prevention. In this context, volunteers have also been encouraged to contribute as partners in the support and follow-up of individuals with mental health issues. Although mental health services were primarily hospital-based in the past, there has been a shift in recent decades towards community-based care with support from specialist services. The aim has been to foster a respectful partnership between patients, families, and professionals, including a commitment to increasing family involvement and providing greater support to family members. Despite the recommendation for family-focused practice, we believe that health professionals still prioritise their alliance with the patient as their foremost responsibility. In this article, we advocate for an enhanced emphasis on family-focused approaches and underscore the importance of utilising knowledge-based family-focused practice models.

## Introduction

Our research network group has explored various models for family-focused practice within mental healthcare, as evidenced by our research endeavours. The research network has specifically examined perspectives related to families and family members of adults and children with mental health conditions, including children as relatives, health professionals, mental healthcare services, and voluntary activities in a caregiving context. The research focus of our network encompasses various aspects related to family members and carers within mental healthcare. These include family members’ experiences of support, everyday life, alienation, burden, stigma, the quality of mental healthcare, perceived mental healthcare, family involvement, family functioning, psychological distress, and well-being. Additionally, our research explores the experience with various interventions, such as psychoeducation (Hansson et al., 2022), assertive community treatment (ACT) (Weimand et al., 2018), resource group assertive community treatment (RACT) (Sjöström et al., 2021; Weimand et al., 2024), family-centred support conversations (FCSC) (Moen et al., 2021; Aass et al., 2020), and voluntary activities (Andfossen, 2020). Our collective endeavour is to illuminate, develop, and deepen the understanding of the family’s situation when affected by mental health conditions. We contend that the prevailing individual-centric approach in mental healthcare falls short in today’s complex landscape. Consequently, we advocate for spotlighting the family perspective to enhance the quality and sustainability of care and services.

In this article, drawing from international and original research, as well as established models and national strategies, we articulate a research network position that emphasises the significance of family-focused practice in mental healthcare while also considering voluntary care efforts in this field in Norway and Sweden. Our ambition is to contribute knowledge to the overall quality of mental healthcare and seek answers to the question, “Where are we on the road towards family-focused practice in mental healthcare?”

## Background

Over the past decade, there has been a gradual shift away from the traditional model of mental healthcare, which primarily focused on individual treatment, towards a more family-focused practice. This paradigm shift has gained traction and momentum in mental health services across Western countries (Allchin et al., 2021; Foster et al., 2016; Nicholson et al., 2015; Shah-Anwar et al., 2019; Tuck et al., 2023). Despite robust evidence, the implementation of family interventions in mental healthcare services continues to present a challenge, in this case often secondary to resource constraints (Allchin et al., 2021; Killaspy et al., 2022; Tuck et al., 2023).

Families perform significant responsibilities and care tasks towards the patient and thereby form a central part of society’s complete care resources (Kokorelias et al., 2019), even when family members do not live with the person suffering from mental health issues (Johansson et al., 2010). From the perspective of the patient, the support of the family can also be of great importance during the process of recovery (Wood & Alsawy, 2018).

There exist several distinct definitions of the concept of ‘family’ – e.g., “a self-identified group of two or more individuals whose association is characterized by special terms, who may or may not relate by bloodlines or law, but who function in such a way that they consider themselves to be a family” (Whall, 1986, p. 241), or, to put it more simply, “family is who they say they are” (Wright & Leahey, 2005, p. 60). The latter corresponds to Foster et al. (2016) and Osher & Osher’s (2002) concept of family, where a family, and who is included in the family, is defined by its members. Moreover, family can be conceptualised as a system in which one family member’s health and well-being affect other family members (Hanson, 2005). Recognising that mental health issues extend beyond the individual to encompass the entire family necessitates a fundamental change in healthcare services, which underscores the importance of including families throughout the process of treatment and follow-up.

Family dynamics encompass the complex web of relationships and interactions within a family. These dynamics are influenced by various factors, including the family’s cultural background, values, and beliefs. They shape how family members communicate, express emotions, and fulfill roles within the family system. No single theory, model, or conceptual framework can fully encapsulate the intricate interplay among these elements. Consequently, health professionals must acquire a comprehensive understanding of diverse theoretical perspectives as these perspectives act as navigation tools for assessing and intervening with families.

There is often a gap between the delivery of care, requirements, strategies, and guidelines, which can result in hindering the improvement of the quality of mental healthcare (Rebello et al., 2014). The decentralisation of mental healthcare and the scarcity of human and institutional resources pose significant challenges to the quality of mental healthcare (Kilbourne et al., 2018). However, family-focused practice can act as a link to improve collaboration and increase the quality of care among patients, families, and health professionals (Waller et al., 2019).

In recent years, interest in improving the quality of care has increased (Kilbourne et al., 2018), and several efforts have been made to improve mental healthcare – primarily in the development of more effective treatments (Clark et al., 2018), but also by using measuring instrument as a tool for improving the quality of care (Kilbourne et al., 2018). Mental health care is often required to report their outcomes according to standardized core measures and conduct quality improvement activities (Weinmann et al., 2007). Quality of care has been highlighted in several legal rules and regulations. In Sweden, for example, the regulation “Quality Systems in Health and Medicine Care” state that healthcare services should have quality systems for planning, performing, evaluation and improve the care given (National Board of Health and Welfare (2025):12). Care providers have a responsibility to provide high quality care and should therefore make efforts to assess the quality of the care provided. (World Health Organisation, 2006).

Quality of care is a multidimensional concept and may vary depending on the perspective (Donabedian, 1980). It is, therefore, essential to include the patient, family, and health professionals when defining the concept of quality of care, as they all have unique information about the care. Hence, their views of what is important in care delivery are valuable. Schröder (2006) states that providing good quality care needs resources in the form of competent and respectful professionals willing to cooperate with the patient and the family. Schröder (2006) further contends that quality of care in mental healthcare is measured in terms of dignity, security, participation, recovery, and the environment viewed from the perspectives of patients, family members, and health professionals.

### National Strategies for Involvement in Caregiving

Mental healthcare in Norway and Sweden is organised at national, health-region, and municipality/community levels. Since the mid-1980s, local community mental healthcare has aimed to address the decline in the number of hospital beds available for individuals with mental health issues (National Directorate of Health, 2014; SOU (2006):100). Home-based services prioritise the fostering of a respectful partnership among patients, family, and health professionals. The aim is to enhance family involvement and support family members substantially (Borg & Kristiansen, 2008; Ministry of Health and Care Services, 2009; National Directorate of Health, 2014).

The mental healthcare service in general is supposed to provide support, information, advice, and necessary training to persons who suffer from mental health issues. Additionally, it should offer training, support, and guidance to relatives who have particularly burdensome care responsibilities (Ministry of Health and Care Services, 1999, 2001, 2011; Ministry of Labour and Social Inclusion, 2010). The aforementioned national guidelines describe the rights of families and the duties of the health and care service and provide recommendations for good practice. The guidelines cover all groups of family members, regardless of the patient’s diagnosis, and include family, elderly, adults, youth, and children as family. Although the legislation and national guidelines in Sweden and Norway outline health professionals’ responsibilities in supporting and collaborating with families, they provide less specific guidance on family inclusion.

In Norway, a national relative strategy has been established for the period 2021–2025 (Ministry of Health and Care Services, 2020). The primary goals of this strategy include recognising family members as valuable resources, adopting a holistic approach to support their ability to lead good lives, and ensuring that children are not burdened with caregiving responsibilities within the family or beyond. Additionally, national guidelines for all groups of family members, regardless of the patient’s diagnosis, were released in Norway in 2017, further revised in 2019 and last updated in 2024 (National Directorate of Health, 2017). These guidelines highlight how to involve and support family members in relation to health and care services and give recommendations for good practice.

In Sweden, the National Carer Strategy (Ministry of Social Affairs, 2022) aims to better meet the needs of carers within the healthcare and social services. The strategy’s main goals are to meet the needs for interventions for patients, family members’ need for participation and information, and their need of support. The strategy intends to enhance the family members’ perspective within healthcare and social services at the municipal level, thereby ensuring more personalised and equitable support for family members across the country. The foundational principle of this strategy is that family members’ involvement and contributions should always be voluntary. The primary target group for this initiative includes adult family members who care for or support individuals with chronic illnesses, elderly family members, or those with disabilities.

Moreover, in this context, volunteers are encouraged to participate as caregiving partners (Ministry of Culture, 2018; Ministry of Health and Care Services, 2013; NOU, 2011: 11). Different types of volunteers are identified in caregiving (Andfossen, 2016). Organised volunteers operate within voluntary organisations, while so-called super helpers operate both as informal caregivers and organised volunteers – in Norway, the latter group contributes twice as much as organised volunteers in care work (Andfossen, 2019).

### Models and Practices with a Family-focused or Voluntary Perspective

In the following section, we will present a selection of evidence-based models and practices with a family-focused perspective, such as psychoeducation, community-based treatment and recovery programmes, and children and parental models, as well as voluntary organisational activities that have been implemented in Sweden and Norway. The target group is families with dependent children and/or adult family members living with mental health conditions. Notably, these models vary in their approaches: some focus on the individual/patient, whilst others emphasise services and strategies to enhance knowledge and involvement.

Family-focused practice (FFP) acknowledges the strengths and vulnerabilities of individual family members and the family and aims to support family choices and collaboration in treatment (Foster et al., 2016). Such practices may be carried out by working directly with the patient suffering from a mental health condition and/or with their children, partner or other family members (Foster et al., 2012). Timely provision of FFP can help to reduce subjective and objective burdens for family members; improve mental health literacy; support family relationships, children’s well-being, and family recovery; and have a preventive effect for intergenerational mental health conditions (Foster et al., 2012). FFP includes both *family-related* and *family-centred* perspectives. *Family-related care* involves viewing the family as a contextual backdrop, with the individual as the primary focus for assessment and intervention. Family involvement varies in this approach. In contrast, *family-centred care* considers the family as a cohesive unit and focuses on the inclusion of family members simultaneously. Family-centred care actively engages significant others within the patient’s family system and recognises their perspectives and knowledge in the planning and delivery of care and support (Shajani & Snell, 2023). An example of this can be seen in Iceland, where family nursing intervention models (Gisladottir & Svavarsdottir, 2017; Gisladottir et al., 2017; Sveinbjarnardóttir et al., 2013; Svavarsdottir et al., 2019), which view the family system as the unit of care, have been developed and implemented in nursing practice at the institutional level since 2006 (Svavarsdottir, 2008).

In the following, the basis for family-focused practice that includes the adult family members is described, as well as a brief review of such models: *Family psychoeducation* and *community-based treatment and recovery programmes* such as Assertive community treatment (ACT), Flexible assertive community treatment (FACT), and Resource group assertive community treatment (RACT).

*Family psychoeducation* includes a group of models used in connection with a spectrum, which ranges from different mental health issues to severe mental health conditions. The structure of family psychoeducation may vary, such as interventions focusing on the family or individual family members as consumers, parents, siblings, friends, etc., or interventions regarding a single-family or multi-family group (McFarlane et al., 2003). Family psychoeducation includes content concerning illness, medication, and treatment management; the coordination of services; paying attention to all parties’ expectations, emotional reactions, and distress; assistance with improving family communication; structured problem-solving and instruction; implementing individualised coping and rehabilitative strategies; expanding social support networks; and explicit crisis planning with professional involvement (McFarlane, 2016). Family psychoeducation is mentioned in various treatment guidelines for schizophrenia and other serious mental health conditions (McFarlane, 2016). The Swedish and Norwegian national guidelines for care and support in schizophrenia and similar conditions (National Directorate of Health, 2013; The National Board of Health and Welfare, 2018) emphasise the importance of including family psychoeducation in the treatment of patients with psychosis, with the first illness episode treated as the highest priority. Research from both Sweden (The National Board of Health and Welfare, 2022) and Norway (Hestmark et al., 2021) indicates that psychoeducation should be offered to families more frequently. The quality of family psychoeducation is consistently high, but far too few families receive this evidence-based treatment (Hansson et al., 2022, 2023; Hestmark et al., 2021), which, when provided, results in decreased relapse and rehospitalisation rates, reduced burden on families, increased knowledge of the disorder and the mental health services, improved ability to solve problems, better self-care, and improved quality of life (Lyman et al., 2014).

*Community-based treatment and recovery programmes* such as assertive community treatment (ACT), flexible assertive community treatment (FACT), and resource group assertive community treatment (RACT) are models that are currently in use (Nordén et al., 2012a). These models are consistent with a family-related care perspective consisting of multidisciplinary professional teams. The treatments and recovery programmes are carried out in the patient’s own environment rather than in a healthcare setting. An important point is that the RACT team consists of not only professionals but also the patients family members (Nordén et al., 2012b), and seeks to ensure that clients and family members, and their informal (i.e., family, friends, etc.) and formal (i.e., social worker, psychiatrist, peer worker, etc.) networks will be involved in routine services and will become collaborative partners in the treatment and recovery process (Tjaden et al., 2021). In the RACT model, the resource group is central and becomes the platform for facilitating the collaboration between the patient and their informal and formal networks. Members of the resource group are appointed by the patient and include the case manager, the psychiatrist, and significant others. During the meetings, the patients’ own personal development goals and wishes are discussed, and plans for treatment and recovery are made jointly. If possible, the patient is the leader of the resource group. The RACT approach also includes family psychoeducation (Berglund & Borell, 2019). Experiences from research regarding, for example, RACT appeared in a meta-analysis by Nordén et al. (2012b), where care according to the RACT method was associated with reduced disease symptoms, improved functional level, and increased well-being in clients with psychosis.

Concerning the point of view of the family members, two studies investigated their experiences of encounters with services in the contexts of the ACT method (Weimand et al., 2018), and the RACT method (Sjöström et al., 2021; Weimand et al., 2024). The results in both studies showed that the participants largely experienced a positive approach from the health professionals, and only to a small extent felt alienated from their family members’ healthcare.

In the following, the basis for family-focused practice that includes the family´s children and young people, as well as parents are described, as well as a brief review of five such models: *The* f*amily talk intervention*, *Let’s talk about children*,* The family model*,* Child talk*,* and Engage*,* Assess*,* Support and Educate* (*EASE).* As children’s and young people’s mental health and well-being are closely linked to those of their parents, models and methods that include their family’s perspective strongly emphasise the need to include the parents. (Falkov, 2012).

*The family talk intervention* (Eklund et al., 2022; Myers et al., 2023)^1^ was developed to prevent children from developing mental health issues, in particular depression, and to strengthen family relationships.

*Let’s talk about children* (Solantaus et al., 2009) is a method for professionals to have structured conversations with parents about parenting and children’s needs related to parental mental health conditions During the conversations between clinicians and parents, the parent’s mental health conditions are explored about the well-being of children (Solantaus et al., 2009).

*The family model* (Falkov, 2012) focuses on both the family’s strengths and challenges. The main purpose is to develop a family plan that supports the needs of family members (Falkov, 2012).

*Child talk* (Reedtz et al., 2012) intervention consists of two to three conversations that involve both parents and children. Its purpose is to support children by providing parents with guidance in their role as caregivers (Weimand et al., 2024).

*EASE* (Foster et al., 2019) is a practice framework that aims to strengthen recovery for persons with mental health issues who are parents, as well as to strengthen nurses’ and other clinicians’ capacity to address and support family recovery concerning parental mental health issues (Foster et al., 2019).

In the following, we will describe *the perspective of voluntary organisations*. Voluntary organisations contribute with different activities for their own members and arrangements that are facilitated for all citizens in a local or regional area, including self-help groups (Andfossen, 2020; Jegermalm et al., 2018). As a citizen, family/informal caregiver or an organised volunteer caregiver, a person might participate in both, either as a contributor or as a receiver. The Norwegian national guidelines (National Directorate of Health, 2017) state that health services should provide family members with information about relevant peer-support groups/NGOs. The interventions are frequently grounded in evidence-based practices, and studies have shown that participation in these groups can positively impact carers’ well-being (Ewertzon et al., 2018). Furthermore, a significant advantage of organisations implementing evaluated, evidence-based support interventions is their potential to be spread beyond the non-profit sector and contribute to regional mental healthcare services. For instance, the Self-Harm and Eating Disorder Organization (SHEDO) in Sweden has successfully disseminated support groups/interventions across multiple regions. They have also established an idea-borne open partnership (IOP) in collaboration with regional and municipal authorities in Sweden. Similarly, in Norway, Mental Health Carers Norway (LPP) describes itself as “a driving force in strengthening mental health care through improved communication between people with mental health conditions, families, therapists, and relevant authorities at municipal and national levels”. They organise self-help groups at local meeting areas throughout Norway (National Association for Relatives in Mental Health, 2024).

## Future Perspective

An emerging consensus highlights that the provision of quality healthcare requires an understanding of the interdependencies among various components of a system, including the family system, and recognition of the relationship between an individual’s health and their environment and context (Dolansky & Moore, 2013). Crucially, the essence of quality care lies in the entire process, with the family unit undeniably playing a vital role (Donabedian, 1988). Consequently, mental healthcare services fall short of quality expectations unless they actively involve and respect the patient’s family members as caregiving partners (MacNeil & Jaggers, 2013), which is in alignment with the principles of family-focused practice (Bell, 2011) and as reflected in national policies and strategies. Furthermore, it is the responsibility of health professionals to ensure high-quality care to families (Schröder et al., 2007).

### Prerequisites and Challenges to Implementing a Family-focused Practice

Below we describe prerequisites and challenges which we have identified to be of importance when focusing on implementing a family focus practice, such as: Adherence to national strategies, National education strategies, Implementation of family intervention models, Health professionals’ approach, and Voluntary organisations.

Regarding the adherence to national strategies, it is encouraging that the positive outcomes associated with family-focused care internationally have led to a shift in policy and practice, which prioritises family-focused practices within adult mental healthcare services (Reupert et al., 2022; Shah-Anwar et al., 2019). In Norway and Sweden, the significance of supporting family choices and collaboration is enshrined in legislation, national guidelines, and scientific literature. However, it surprises us that, despite robust and expanding evidence for family psychoeducation across multiple continents and diverse diagnostic and demographic populations (Lucksted et al., 2012), many families still have limited or no access to an effective version of family psychoeducation (Hestmark et al., 2021). Similarly, although RACT has proven effective, its implementation in clinical practice and family psychoeducation could be more widespread (The National Board of Health and Welfare, 2022). The underutilisation of family-focused practice may be attributed to specific barriers in implementing family psychoeducation within mental healthcare, as well as more general challenges in translating evidence-based treatment into routine clinical practice. Evidence suggests that family psychoeducation is gaining popularity, which may be due to its perceived feasibility for delivery (Killaspy et al., 2022). Even though a family-focused perspective is mentioned in national strategies, it is important that it also includes such a perspective in national education strategies.

Further, National education strategies, like the Swedish national education plan for nursing and specialist nursing education, stipulate that, for the degree, students must demonstrate knowledge and skills independently, and in collaboration with the patient and family members, identify care needs and develop a care plan. Additionally, students must demonstrate a professional approach toward patients and their family members (Ministry of Education, 1993). The national guidelines for nursing education in Norway specify that nurses must be capable of planning and interacting with patients and their family members based on respect, co-determination, and integrity. Moreover, they must collaborate with patients and family members to prevent and resolve conflicts. Furthermore, nurses should be able to plan and implement quality improvement and service development in collaboration with patients and family members. Finally, nurses must be proficient in using technology and digital solutions to support the resources, coping abilities, and participation of patients and their family members (Ministry of Education and Research, 2019).

Notably, it is a step in the right direction that nurses in Norway and Sweden, through master’s and doctoral courses at the Norwegian University of Health and Science (NTNU) and Linnaeus University in Sweden, are theoretically and practically trained in using the Calgary family assessment model (Shajani & Snell, 2023). However, although national strategies and national education strategies advocate family-focused practice, intervention models and family assessment models have been implemented to a limited extent in adult mental healthcare in Sweden and Norway (Clausson & Berg, 2008; Moen et al., 2021; Aass et al., 2020).

Concerning the implementation of family intervention models, health professionals play a crucial role in supporting families through, e.g., personal and emotional support. This support promotes effective family functioning and contributes to the overall well-being of patients and their families (Falkov et al., 2020; Shajani & Snell, 2023, Aass et al. 2021; Aass et al. 2022).. Family intervention models such as family psychoeducation, skill training, counselling, family meetings, and the acknowledgement of strengths can be crucial to contribute to the well-being of patients and their families. Evidence indicates that mental healthcare services still largely operate within traditional structures and have been slow to adapt to new policies and strategies (Montenegro et al., 2023). Family members of patients with mental health conditions often experience emotional suffering long before their relative is first admitted to psychiatric care, and their caregiving efforts are frequently rejected by the patient. These families face complex situations that affect their behaviour and expressed emotions, and that need to be understood within this context (Phillips et al., 2023).

Moreover, compelling evidence suggests that involving family members in the treatment process is highly beneficial and can be a game-changer. This leads to improved treatment adherence (Cahaya et al., 2022; Cañas et al., 2013), reduced burden of care on family members [name deleted to maintain the integrity of thereview process], fewer hospital admissions, with shorter stays (Pfammatter et al., 2006), all of which are significant outcomes associated with family involvement. A vast majority of family interventions show effectiveness in preventing relapse even at follow-up observations longer than 12 months (Barlati et al., 2024).

Mental health conditions, parenting and children’s development are strongly connected (Falkov et al., 2020). It is crucial to emphasise that children and young people need opportunities to discuss their situation, and they require information about the illness and its consequences as well as practical support at home (Ruud et al., 2015). Health professionals, however, often believe that the families receive more support at home than they actually do (Ruud et al., 2015). This discrepancy highlights the need for family-focused practices to include the experiences and voices of young family members. Such practices not only support the young persons but also enhance parents’ skills and understanding of their children’s needs. Notably, in child mental healthcare, the family model is implemented in both Norway and Sweden through a top-down approach (Linderborg et al., 2022, 2024; Weimand et al., 2017).

A further prerequisite and challenge for implementing family-focused practice is to ensure the health professionals’ approach towards such a perspective. Despite the recognised importance of family for individuals with mental health conditions (Foster et al., 2016, 2019; Aass et al., 2021), daily clinical practice often prioritises individual patient treatment and follow-up and is frequently aligned with the patient’s own preferences (Skundberg-Kletthagen et al., 2020). We emphasise that a trusting alliance between health professionals and families should also be considered essential for patient care [name deleted to maintain the integrity of the review process]. This, in our opinion, requires health professionals who are equipped with curiosity and knowledge about the reciprocity between mental health conditions and family functioning. We assert that a sustainable approach from health professionals regarding family-focused practice is important to ensure that families receive high-quality mental healthcare (Allchin et al., 2021; Linderborg et al., 2024; Moen et al., 2021; Aass et al., 2020). Hence, we advocate for quality improvement efforts, including increasing knowledge about the importance of family-focused practice (Foster et al., 2016), as well as the development of continuing education and training programmes for health professionals to enhance their understanding of and supportive attitudes towards mental health conditions (Moen et al., 2021; Aass et al., 2020). Based on the need for a broadened approach to the treatment and support of people with mental health conditions, an important component in the implementation of family-focused practices is to provide the personnel with the necessary education and skills.

Our research findings illuminate the persistent influence of outdated assumptions and cultural shifts on the concept of family, particularly within adult mental healthcare practices (Moen et al., 2021; Skundberg-Kletthagen et al., 2020). This underscores the evolving nature of our understanding of ‘family’ and its implications for clinical practice. Notably, the manner in which health professionals define and incorporate the concept of family into their practice – whether as a contextual factor or an integral unit of care – can significantly impact treatment outcomes (Foster et al., 2016).

It is crucial to recognise that the conceptualisation and implementation of family-focused practices may vary based on the specific setting or context, such as child or adult mental health services (Foster et al., 2019). However, irrespective of context, mental healthcare professionals hold the key to empowering family members with the necessary skills and confidence. This not only enhances their ability to cope but also ensures safe and effective patient care (Phillips et al. 2023; Shajani & Snell, 2023). Based on the need for a broadened approach to the treatment and support of people with mental health conditions, an important component in the implementation of family-focused practices is to provide the health professionals with the necessary knowledge and skills (Foster et al., 2016).

A further challenge in achieving the breadth of a family-focused practice is to see the importance of collaboration and the gap between health care services and voluntary organizations. Considering the imminent shortage of professional carers in mental healthcare, it is crucial to facilitate and deliver mental healthcare services differently to ensure sustainability. National governing documents suggest the involvement of family members and volunteers as a solution to this challenge (Ministry of Health and Care Services, 2013; NOU, 2011: 11; NOU 2023: 4). Many family members of persons with mental health conditions often find themselves in need of information about the illness, better understanding of the healthcare system and opportunities to connect with others in the same situation. Commonly, they express a need for support in their roles as family members (Ministry of Social Affairs, 2022). Hence, in addition to family involvement, voluntary organisations play a crucial role in providing essential support groups for family members as caregivers. These organisations often adopt a holistic perspective that includes both carers and the individuals with a mental health condition. Many organisations facilitate groups where carers can connect with others in similar situations, such as other parents or siblings. These groups are typically led by fellow carers or professionals. Our experience is that in recent years, collaboration between healthcare services and voluntary organisations in Sweden and Norway has developed. It is a challenge in the future to further develop collaboration so that the gap between organisations is reduced and provides opportunities for further development of a breadth of family-focused practices.

The overall question for this article was, “Where are we on the road towards family-focused practice in mental healthcare?” Based on our experience and knowledge presented in this article, the development of family-focused practice in Sweden and Norway is partly in progress but still limited. Even if there are many prerequisites for such practice, there are still many challenges to prevent the development from stalling. Prerequisites and challenges, such as family-focused practice, is mentioned in *national strategies* and in *national nursing education strategies*. However, implementation of *family intervention models is limited*, and the health professionals often prioritise the *individual patient perspective*. To broaden the approach to a family-focused practice is to provide health care services and professionals with the necessary knowledge and skills. A further prerequisite and challenge in achieving the breadth of a family-focused practice is to develop collaboration between health care services and voluntary organisations so that the gap between the organisations will be reduced.

We still interpret family-focused practice in Sweden and Norway as a Fresh Focus perspective, where there are several prerequisites and challenges to monitor to ensure implementation in clinical practice, and further research is important to ensure that development does not stagnate.

Based on the current body of knowledge, we urgehealth professionals and providers to enhance the implementation and accessibility of evidence-based, family-focused models in mental healthcare. Despite the known barriers, it is imperative to acknowledge that mental health conditions is a family affair, which necessitates a focus on the family as a unit. As an approach, it better meets the current challenges in healthcare services, where patients are mostly followed up at home, not in a hospital. To effectively implement family-focused practices, all members of a clinical team should receive training and regular supervision, employing a ‘whole-team approach’. Developing a clear structure for the intervention/model while ensuring flexibility to accommodate individual needs may enhance the perceived quality of care by families. This is crucial, as previous studies have demonstrated that patients and family members who are offered participation and involvement in care report the quality of care as superior. Concerns regarding privacy, power dynamics, fear of negative outcomes and the necessity for an exclusive patient–professional relationship will inevitably arise. Addressing these concerns through open, non-judgemental communication can facilitate the establishment of a therapeutic alliance between health professionals, families and patients. This may elucidate why family interventions – despite their robust evidence base and inclusion in nearly all policies and guidelines – are so poorly implemented in routine practice. The identified requirements may be challenging, as family-focused practices must be embraced by the entire organisation and integrated into work routines to be effectively implemented.

## Footnotes

1 Beardslee’s Family Talk Intervention (sbu.se).
